# Trust in AI-assisted health systems and AI’s trust in humans

**DOI:** 10.1038/s44401-025-00016-5

**Published:** 2025-03-28

**Authors:** Madeline Sagona, Tinglong Dai, Mario Macis, Michael Darden

**Affiliations:** 1 Bloomberg School of Public Health, Johns Hopkins University, Baltimore, MD USA; 2 Hopkins Business of Health Initiative, Johns Hopkins University, Washington, DC USA; 3 Carey Business School, Johns Hopkins University, Baltimore, MD USA; 4 School of Nursing, Johns Hopkins University, Baltimore, MD USA; 5 Berman Institute of Bioethics, Johns Hopkins University, Baltimore, MD USA

**Keywords:** Health care economics, Information systems and information technology, Science, technology and society

## Abstract

Artificial intelligence (AI) is reshaping healthcare, promising improved diagnostics, personalized treatments, and streamlined operations. Yet a lack of trust remains a persistent barrier to widespread adoption. This Perspective examines the web of trust in AI-assisted healthcare systems, exploring the relationships it shapes, the systemic inequalities it can reinforce, and the technical challenges it poses. We highlight the bidirectional nature of trust, in which both patients and providers must trust AI systems, while these systems rely on the quality of human input to function effectively. Using models of care-seeking behavior, we explore the potential of AI to affect patients’ decisions to seek care, influence trust in healthcare providers and institutions, and affect diverse demographic and clinical settings. We argue that addressing trust-related challenges requires rigorous empirical research, equitable algorithm design, and shared accountability frameworks. Ultimately, AI’s impact hinges not just on technical progress but on sustaining trust, which may erode if biases persist, transparency falters, or incentives misalign.

## Introduction

Artificial intelligence (AI) is reshaping medicine, promising to improve diagnostics, personalize treatments, and streamline operations. Yet despite the boom in medical AI—1016 AI devices have been cleared by the U.S. Food and Drug Administration for clinical use as of September 2024^1^—hesitancy about routine use of AI persists. This hesitancy extends beyond technical limitations and centers on trust—not just in AI tools but also in the creators underlying them^2^. As one colleague observed, “Every panel discussion about AI and health eventually became a trust panel”. For AI to deliver on its promise, trust must underpin its use at every level of health systems.

Trust involves vulnerability and uncertainty. It means relying on others with the expectation that they will act competently and in good faith^3^. According to Luhmann (1982), trust creates the space for risk-taking, enabling cooperation and decision-making in the face of uncertainty^4^. Similarly, Gambetta (2000) describes trust as a subjective probability—the likelihood, as perceived by one party, that another will act in a way that is beneficial or at least not detrimental^5^. Building on this idea, in healthcare trust allows patients to accept vulnerability in exchange for care, gives providers the confidence to make decisions, and shapes how institutions interact with communities.

AI is reshaping the way trust works in healthcare. Unlike traditional medical tools, many AI tools operate as “black boxes,” meaning the logic behind their outputs is unclear. This raises thorny questions: Can people trust a tool when they don’t understand how it works? Is transparency essential, or are other forms of validation sufficient?

But AI also strains an already fragile trust dynamic. In many cases, patients do not interact with AI directly; they must trust providers to adopt AI wisely and use discretion in following its recommendations. Just as patients trust providers to competently use MRI machines without understanding their inner workings, AI requires a similar, yet more complex, delegation of trust—one complicated by existing skepticism toward healthcare institutions and concerns over how AI is deployed in practice.

Medicine has always required trust in the unseen. Consider acetaminophen (Tylenol). Despite its widespread use, its precise mechanisms of action remain unclear. Yet trust in its safety and effectiveness has been built over decades of consistent use and experience, reinforced by the rigor of clinical trials and regulatory oversight. Similarly, AI may not need to be fully explainable to earn trust, but it must build a record of “getting it right” through consistent safety, effectiveness, and ethical soundness.

Yet “explainable AI” remains a policy priority. Most explainability methods rely on a second, simpler model trained to approximate the predictions of the black-box AI. These surrogate models are typically used for post hoc explanations rather than actual decision-making because they are less accurate than the black box itself^6^. Explainable AI, therefore, can offer only an approximation—not true transparency—and as a result is unlikely to improve trust^6,7^. Yet in practice, patients do not need to understand AI directly any more than they understand an MRI scan; what matters is whether providers can interpret and communicate AI-driven insights effectively. If AI enhances providers’ ability to explain and justify decisions, it may build trust even without full transparency.

Explainability is only part of the challenge. Minoritized communities, whose trust in health systems has been eroded by historical inequities, ongoing biases, and outright malevolence, are likely to approach AI with heightened skepticism. These communities have seen how systemic disparities can be perpetuated by the tools meant to serve them, and AI risks exacerbating these inequalities if it is not designed and validated with equity in mind.

This Perspective explores the emerging role of AI in influencing trust in healthcare. It examines the relationships AI affects, the systemic inequities it may reinforce, and its technical underpinnings. It aims to outline a path toward a future where trust in medical AI is earned, justified, and extends to the health systems that deploy it.

## Mapping the web of trust

**Fig. 1:**
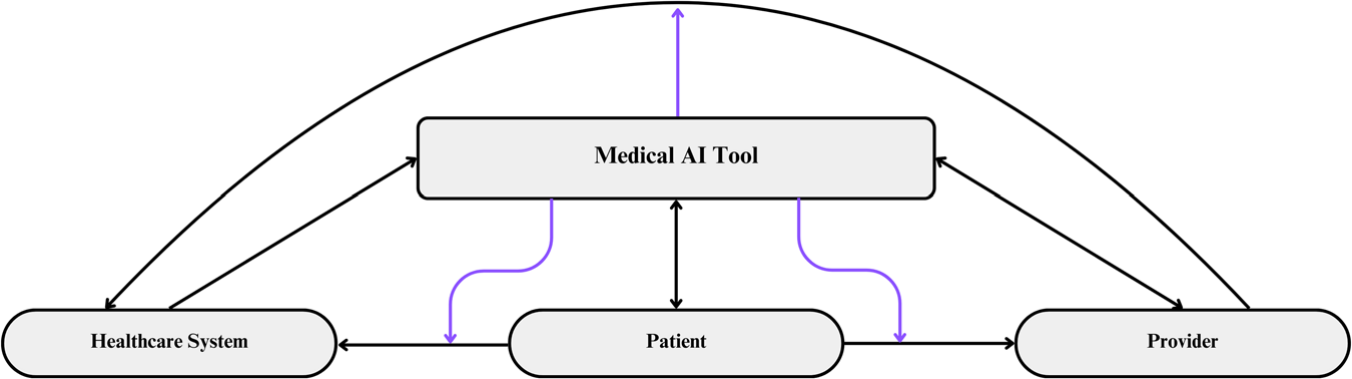
Potential trust relationships at play. This figure illustrates the web of trust between key stakeholders in AI assisted healthcare systems, including patients, providers, AI technologies, and healthcare institutions. Arrows indicate the directionality of trust relationships, highlighting that trust must flow both ways for effective adoption and integration of AI into clinical care.

Trust in healthcare is shaped by interactions between key stakeholders. Its dynamics shift depending on the relationships at play—whether between patients and providers, providers and AI, patients and health systems, or providers and the institutions they serve (Fig. 1). We examine each of these relationships below.

### Patient trust in the provider

Trust is a cornerstone of the patient-provider relationship. Patients trust providers to be competent and committed to their patients’ well-being. Research shows trust improves health outcomes^8–10^. Patients who trust their providers are more likely to seek care, adhere to treatment plans, follow medical advice, and experience better overall health^10–13^. However, AI’s growing role in clinical care may complicate this trust.

For some patients, a provider’s use of AI may convey innovation and better care, strengthening trust. For others, it may suggest a lack of expertise, insufficient human attention or effort, or overreliance on technology^14^, undermining confidence in the provider. This skepticism is echoed in a 2023 Pew Research Center survey, which found that a majority of U.S. adults across demographic groups expressed discomfort with the idea of their healthcare provider relying on AI^15^. Another concern is that AI might not just assist providers but displace their judgment, making patients feel as though their care is being outsourced to a “cookie cutter” algorithm rather than guided by human expertise^16^.

These concerns are intertwined with broader issues of trust and incentives in healthcare. Patients generally understand physicians may respond to financial incentives. Sometimes these incentives align with patient health, as in certain pay-for-performance models that tie reimbursement to risk-adjusted health outcomes. But when they do not, trust can falter. AI raises the question of whether it will create more opportunities for these misalignments, further straining trust. For example, AI-powered clinical decision support tools could be designed to optimize hospital efficiency by prioritizing lower-cost treatments, potentially leading to undertreatment of certain patients. Research is needed to determine how AI affects patient perceptions of their providers, particularly across different demographic and cultural contexts.

AI offers both opportunities and challenges to the relational aspects of care. Topol (2019) argues AI can alleviate the burden of electronic health records (EHRs), enabling more meaningful patient interactions^17^. While AI holds the promise of streamlining administrative tasks like documentation—potentially making healthcare more human, as Topol suggests—efficiency gains in for-profit hospitals may instead be used to increase caseloads, further limiting time with patients and weakening provider–patient connections. This tension can, somewhat ironically, resemble the experience of EHR implementations, which have improved data access but increased documentation time—often at the expense of patient engagement.

Communities such as Black, Hispanic, and Native American populations report lower baseline trust in the healthcare system due to historical injustices and ongoing systemic inequities^18^. For these groups, trust in providers is already fragile, and the introduction of AI could deepen skepticism. Concerns about fairness, transparency, and bias in AI systems are particularly acute, as these tools risk perpetuating disparities if they are not rigorously validated for equity.

### Provider Trust in AI

Trust between healthcare providers and AI differs from interpersonal trust, which often involves assumptions about goodwill and intent. However, the adage “trust people to be who they are” underscores a more practical dimension of trust: predictability. In this sense, trust in AI does not depend on motives but rather on the system’s ability to deliver consistent and reliable results.

Concerns over liability temper provider trust in AI. Price et al. (2019) argue current malpractice law protects doctors who follow the standard of care but not those who deviate from it. This legal structure may discourage physicians from relying on AI beyond a confirmatory role^19^. If AI becomes the standard of care, liability could shift—doctors who ignore AI recommendations might be held accountable for bad outcomes. At the same time, if a physician follows AI advice and harm occurs, it is unclear whether the fault lies with the doctor, the AI developer, or the institution using the tool. These legal uncertainties make providers cautious about adopting AI^19,20^. As Jesse M. Ehrenfeld, former president of the American Medical Association, wrote, “AI is too powerful and too revolutionary to leave questions about liability and governance unanswered”^21^.

Providers may be wary of AI because they fear job displacement and question whether its recommendations align with their clinical expertise. An international survey in 2023 found 38% of radiologists feared being replaced by AI^22^. Notably, increased knowledge about AI tended to reduce fears. Providers may also question whether AI models correctly prioritize clinical factors. For example, in one study of an AI sepsis early warning system, emergency physicians expressed concern that the model overemphasized the importance of blood samples in its risk assessments^23^. Some physicians reported that they would be more likely to trust the tool if they received feedback on cases where it detected sepsis that they had initially missed.

Concerns about surveillance complicate trust in the current political milieu. AI’s ability to record, analyze, and document clinical interactions can make many providers uncomfortable. For example, in states with restrictive abortion laws, if a provider’s conversation with their patient is being recorded and documented by an AI tool, they may not feel comfortable talking freely about all possible healthcare options available to the person considering abortion.

### Patient trust in the healthcare system

Trust in the U.S. healthcare system is low, with a 2023 Gallup poll showing that only one-third of Americans have confidence in it^24^. A recent study found that 60% of Americans have only some or less trust in their physicians and hospitals, as opposed to “a lot” of trust^25^. The use of AI could worsen patient trust in the health system, especially if systems prioritize cost reduction, revenue maximization (e.g., through AI-assisted medical coding), or efficiency over patient outcomes.

Indeed, this misalignment of priorities is already playing out in the health insurance landscape. UnitedHealthcare allegedly used a faulty AI tool with a 90% error rate that denied elderly patients medically necessary coverage. Families of two elderly patients who died due to denied coverage are suing UnitedHealthcare, claiming that the insurance company continues “to deny claims using their flawed AI model because they know that only a tiny minority of policyholders (roughly 0.2%) will appeal denied claims, and the vast majority will either pay out-of-pocket costs or forgo the remainder of their prescribed post-acute care”^26^.

### Provider trust in the healthcare system

Like patients, many providers also perceive healthcare institutions as prioritizing efficiency and profit over values that matter to physicians, such as patient health and quality of care. AI can exacerbate this concern. Providers may worry that AI tools are chosen not for their clinical accuracy or patient-centered design but for their ability to streamline operations or reduce costs. This perceived misalignment can undermine provider trust in both the institution and the AI itself.

Trust may be even more important to providers than it is to patients. Providers are often the first to recognize when AI tools are being used and are uniquely positioned to assess their strengths and limitations. While patients may never realize that an algorithm influenced their diagnosis, providers must engage directly with these systems and decide whether to integrate their recommendations into care. Providers find themselves having to trust both the AI tool itself and the institution that chose to deploy it. When providers are excluded from decisions—whether in the development, selection, or evaluation of these technologies—their trust in both the tool and the institution begins to fray.

To build and maintain providers’ trust, institutions must be open about their AI strategy. They must also demonstrate these tools are aligned with clinical priorities, with a focus on enhancing patient care rather than simply driving efficiency. Without this alignment, providers may view AI as an administrative mechanism rather than a meaningful partner in advancing healthcare.

### Patient Trust in AI

Some have argued that a patient’s trust in a physician by extension implies trust in the tools they use, including AI tools^10^. For example, patients typically trust the provider interpreting an X-ray rather than questioning the reliability of the machine. However, AI differs from conventional technologies in that its outputs are shaped by complex algorithmic layers (including training data quality, developer assumptions, and probabilistic reasoning) that introduce variability and potential bias. Unlike static tools, AI systems evolve based on the diversity and completeness of their data, which can reflect or even amplify systemic inequalities. This makes it challenging for providers to assess when an algorithm might be biased, by how much, and in which direction, further complicating trust.

The potential for bias is not just theoretical. Obermeyer et al. show that a commonly used risk prediction tool assigned sicker Black patients the same risk scores as healthier white patients, diverting resources away from Black patients with greater needs^27^. The bias arose because the algorithm used healthcare costs as a proxy for patient need, inadvertently mirroring systemic disparities in which less money is spent on Black patients even when their health is worse. Careful design can prevent such inequities and promote fairness; in this case, reformulating the algorithm to prioritize clinical need over cost can mitigate the reported bias.

Inequities perpetuated by AI may be particularly pernicious because, on the surface, AI outputs appear objective^28^. After all, unlike humans, AI systems cannot harbor racist views. But the humans who create the models can. On top of that, humans train and validate AI tools using data derived from a medical system that has historically^29–33^ and continues^34–36^ to discriminate against minoritized groups, and those practices are reflected in and become a part of the data^28^. If AI systems are not carefully designed and regularly audited for bias, they risk reinforcing and legitimizing discriminatory medical practices under the guise of neutrality.

Patient trust cannot be demanded or coerced. It must be the earned result of a system that consistently puts their well-being ahead of profit or convenience. For patients to trust AI, AI needs to be *trustworthy*. Trustworthiness entails demonstrating competence, honesty, and concern for the trustor’s well-being^3,5^. Several countries^37–39^ have issued guidelines articulating what trustworthiness entails in the context of AI. These guidelines prioritize safety, privacy, transparency, accountability, and avoiding discrimination^40^. If these conditions are met, trust will likely follow.

### Healthcare system trust in AI

Due to the scoping nature of the “healthcare system,” our discussion on the healthcare system’s trust in AI will be non-exhaustive. “The healthcare system” is used here to refer to hospitals, insurers, and regulatory bodies. Hospital systems will care about whether AI tools can integrate seamlessly into workflows without introducing unintended harm. As with providers, hospital administrators will have to grapple with liability concerns.

Insurance companies widely employ AI tools for marketing, pricing, claims processing, fraud detection, and operational efficiency^41^. Although these applications often focus on administrative tasks rather than direct clinical decision-making, errors in AI—such as faulty claim denials—can significantly undermine trust in insurers. In fact, insurance companies are among the least trusted healthcare institutions in the U.S., tied with pharmaceutical companies^13^. This illustrates that even when AI is used outside of clinical contexts, its reliability remains critical to maintaining stakeholder confidence.

Regulatory bodies such as the U.S. Food and Drug Administration (FDA) determine which tools meet safety and efficacy standards. Whether an AI tool gains approval often hinges on whether regulators deem it safe, effective, and reliable—factors that serve as institutional proxies for trust.

### AI’s trust in human decision-makers

Discussions of trust in AI often focus on whether humans can trust machines. But AI systems also depend on the quality and reliability of human inputs, creating a bidirectional trust relationship^42^. AI does not “trust” in the human sense; it lacks the capacity for subjective judgment about the other party’s intentions. However, AI does rely on humans for data collection, labeling, and oversight.

Operational oversight deepens this interdependence. Providers must decide whether to trust or override AI recommendations, but AI may struggle to contextualize these decisions, misinterpreting deviations as errors rather than expertise. Embedding feedback mechanisms can help AI learn from provider actions and build mutual trust.

Bidirectional trust in human–AI interactions raises accountability questions. If AI fails due to flawed inputs or if providers ignore accurate recommendations, who is responsible? Shared accountability frameworks are vital for fostering mutual trust.

Future research should explore how AI systems can model and incorporate trust in human inputs. Incorporating principles of bidirectional trust—where both humans and AI adapt and respond to one another—will be key to developing collaborative systems that improve healthcare outcomes while maintaining reliability and fairness.

## How trust shapes the way we seek care

Throughout this discussion, we have emphasized that trust in AI extends beyond its technical accuracy. Whether patients seek care at all—and how they engage with healthcare providers—is deeply shaped by trust. Darden and Macis^13^ offer a care-seeking behavior framework that provides a useful lens for investigating this dynamic. According to this model, care-seeking is driven by three factors: the expected benefit of care, the expected cost of care, and the perceived quality of interactions between patients and healthcare providers (or other parts of the system). Trust influences all three, shaping patients’ expectations of outcomes and the quality of their interactions, and ultimately influencing their willingness to seek care.

AI can shift these trust dynamics by altering how and when people seek care, changing their expectations about the benefits, costs, and quality of interactions with providers. For example, AI could increase trust in care by improving diagnostic accuracy, decrease trust if it undermines personal connections, or change perceptions of cost by making processes more efficient. The effect of AI will also vary by application. Administrative AI, such as automated insurance claims processing, will affect trust differently than AI used for diagnostics or treatment recommendations.

Darden and Macis provide empirical evidence that trust is strongly associated with care-seeking behavior, even after controlling for income and education^13^. Similar findings have been reported in prior research^10–12^. This suggests the need to incorporate trust into the develop-deployment lifecycle of AI tools. By measuring and accounting for trust at every stage, AI tools can be improved in both functionality and acceptance by providers and patients. Going forward, developing methods to quantify trust and disentangle its multiple effects will be essential to building AI systems that improve care while maintaining patient trust.

## Conclusion

Kenneth Arrow, a Nobel laureate in economics and a pioneer of health economics, observed, “Trust is an important lubricant of a social system. It is extremely efficient; it saves a lot of trouble to have a fair degree of reliance on other people’s word. Unfortunately, this is not a commodity which can be bought very easily. If you have to buy it, you already have some doubts about what you have bought”^43^.

In this Perspective, we argue trust is built differently in each relationship: between patients and providers, providers and technology, and institutions and their stakeholders. Trust is also bidirectional; people must trust AI to perform reliably, while AI relies on the quality of human input. A future in which AI meaningfully improves health outcomes will not be achieved through technological advancements alone. It requires shared accountability, thoughtful governance, and an unwavering commitment to equity. If trust is earned and cultivated at every stage of implementation, AI can become a partner in medicine rather than a source of skepticism.

## Data Availability

No datasets were generated or analysed during the current study.
